# Immune-checkpoint inhibitor-associated grade 3 hepatotoxicity managed with enteric-coated budesonide monotherapy: A case report

**DOI:** 10.1097/MD.0000000000029473

**Published:** 2022-08-05

**Authors:** Gerasimos Eleftheriotis, Elias Skopelitis

**Affiliations:** a 2nd Department of Internal Medicine, General Hospital of Nikaia-Piraeus “Agios Panteleimon,” Athens, Greece.

**Keywords:** budesonide, case report, hepatitis, immune-checkpoint inhibitor, ipilimumab

## Abstract

**Rationale:**

The introduction of immune-checkpoint inhibitors (ICPI) in recent years has changed the natural course of many neoplasms. However, patients receiving these medications may present immune-mediated adverse events; management includes temporary or permanent cessation of treatment and corticosteroids, occasionally combined with other immunomodulators. Such immunosuppression, however, also has numerous adverse events and even if it is effective in controlling toxicity, it delays immunotherapy reinitiation, as current evidence requires dose tapering to ≤10 mg prednisolone equivalent before rechallenge. Enteric-coated budesonide is a corticosteroid formulation acting primarily to the intestine and liver, as a result of its extensive first-pass hepatic metabolism.

**Patient concerns:**

A 76-year-old woman treated with ipilimumab for metastatic melanoma presented with abdominal pain, vomiting, and diarrhea for at least the previous 4 days. Laboratory tests, among others, revealed elevated aminotransferases and C-reactive protein. During hospitalization, the patient also developed fever.

**Diagnosis:**

The patient, after excluding alternative causes of aminotransferase elevation, was diagnosed with grade 3 ipilimumab-associated hepatotoxicity.

**Interventions:**

Budesonide monotherapy was administered; initial daily dose was 12 mg.

**Outcomes:**

Fever subsided after the first dose of budesonide. Aminotransferases returned to normal-near normal approximately 1 month after the first dose of budesonide. After this point, daily dose was reduced by 3 mg every 2 weeks, with no clinical or biochemical relapse.

**Conclusions:**

This case of ICPI hepatitis is, to our knowledge, the first in the literature managed with budesonide monotherapy. Therefore, budesonide may be a potentially attractive option for the management of ICPI-associated liver injury in cases where corticosteroid treatment is necessary due to its safety profile and the potential advantage of faster immunotherapy rechallenge in selected patients without requiring dose tapering, in contrast to systemically acting corticosteroids. Clinical trials should be conducted in the future in order to validate or refute these findings.

## 1. Introduction

Immune-checkpoint inhibitors (ICPI) are monoclonal antibodies targeting immunity downregulators like cytotoxic T-lymphocyte antigen 4 (CTLA-4), programmed cell death 1 (PD-1), and programmed cell death ligand 1. ICPI administration has prolonged survival in many solid tumors like melanoma and renal cell carcinoma without the classic chemotherapy-associated side effects. However, patients can experience toxicities mimicking autoimmune disease because they promote immune system activation. These manifestations can affect multiple organs or only a single organ. Several approaches have been applied, lots of them through extrapolation of data from customary autoimmune disorders. These include temporary or permanent suspension of immunotherapy and usually corticosteroids, either as monotherapy or in combination with other agents if there is no improvement.^[1,2]^

Recent American Gastroenterological Association Clinical Practice Update for ICPI colitis and hepatitis suggests discontinuation of immunotherapy for grade 1 or grade 2 hepatic toxicity, whereas 0.5 to 1 mg/kg/d of prednisolone equivalent can be initiated if grade 2 hepatitis persists after 1 to 2 weeks of delay in ICPI dosing and in patients with symptomatic grade 2 hepatitis.^[3]^ For more severe liver enzyme impairment, prompt administration of 1 to 2 mg/kg/d prednisolone equivalent for grade 3 and 2 mg/kg/d for grade 4 toxicity is indicated.^[3]^ Steroid dosage can be uptitrated and/or mycophenolate mofetil (1000 mg twice a day) can be added to the regimen in grade ≥ 3 nonresponders after 3 to 5 days of corticotherapy.^[1,3]^ Other alternatives to mycophenolate are azathioprine and tacrolimus (50–150 mg/d or 1–2 mg/kg/d [provided serum bilirubin is < 6 mg/dL] and 1–8 mg/kg/d, respectively), following dosing strategies for autoimmune hepatitis (AIH).^[1,3–7]^ Anti–tumor necrosis factor inhibitors should be avoided if possible because they can cause drug-induced liver injury themselves. The aforementioned noncorticosteroid immunosuppressants are also necessary for corticosteroid-dependent patients.^[3]^ Glucocorticoid tapering can be attempted within 4 to 6 weeks, although longer courses of treatment may be required.^[2]^ Nonetheless, it should be noted that toxicity from immunotherapy is a diagnosis of exclusion; all patients should also be evaluated for other causes of elevated liver enzymes.

Enteric-coated budesonide is a synthetic glucocorticoid in a formulation able to deliver the drug to the ileum, where it is absorbed. Then it undergoes extended first-pass metabolism from cytochrome P450 3A4 in the liver to compounds with negligible glucocorticoid activity (16α-hydroxyprednisolone and 6β-hydroxybudesonide).^[8]^ Due to that odd pharmacokinetic profile, it has only been typically used to treat disorders involving organs exposed to the whole drug dose, like inflammatory bowel disease and AIH.

2015 European Association for the Study of the Liver update and 2019 American Association for the Study of Liver Diseases and Greek guidelines for AIH suggest budesonide-containing regimens either as first-line treatment or as an alternative.^[4–6]^ There are data indicating that budesonide combination with azathioprine achieves similar, if not better, remission rates in AIH compared with prednisolone plus azathioprine, although some argue that data of histological response in budesonide-treated patients are lacking.^[9]^ Its reduced systemic absorption (from 9%–12% in healthy individuals to 21% in patients with Crohn disease in the absence of cirrhosis, portosystemic shunt, or concomitant use of cytochrome P450 3A inhibitors) leads to lower incidence of side effects compared with other glucocorticoids.^[8,10]^ In addition, total systemic exposure to the drug after first-pass metabolism is estimated to be 40-fold less than that of methylprednisolone, according to Edsbäcker et al.^[8,11]^

## 2. Case Report

A 76-year-old Caucasian woman presented to the emergency department complaining of abdominal pain, nonbloody, nonbilious vomiting, and nonbloody diarrhea (increase of 3 stools per day over baseline) for at least the previous 4 days. Her medical history included osteoporosis, total thyroidectomy due to thyroid cancer followed by a course of radioactive iodine 13 years ago, cholecystectomy 3 years ago after an episode of acute biliary pancreatitis, and amelanotic melanoma of the left first toe managed initially with surgery and adjuvant immunotherapy with pembrolizumab, after which relapse was noticed, manifesting with metastatic lesions on the subcutaneous tissue of the left thigh and the left external iliac lymph nodes. For that, the patient was started on ipilimumab 3 mg/kg q3 week by her oncology team. She had already received 3 doses, the last 19 days before admission.

Her vital signs on admission included 111 heart beats per minute, axillary temperature 37.2^o^C, blood pressure 112/80 mm Hg, and respiratory rate of 14 breaths per minute. She was completely alert and oriented. Physical examination of the abdomen revealed normal bowel sounds and epigastric tenderness; however, rebound tenderness couldn’t be elicited. Signs of her melanoma were found, too; cutaneous painless nodules as well as hard, nontender left iliac lymph nodes. The rest of physical examination was unremarkable. Her body weight was 66 kg.

Laboratory tests revealed elevated aminotransferases (alanine aminotransferase [ALT] > aspartate aminotransferase [AST]) and C-reactive protein, pyuria, as well as a mild increase of serum and urine amylase (Table 1). Microscopic stool examination showed numerous leucocytes. No parasites were found. Testing for *Clostridioides difficile* toxin and stool antigen (thrice), serum antibodies for *Entemoeba histolytica*, and stool antigens for *Cryptosporidium spp* and *Giardia lamblia* were negative. An abdominal computed tomography without intravenous radiocontrast was performed, with no abnormal findings.

**Table 1:** Laboratory data.

**Variable**	**Reference** **range,** **adults**	**On** **admission**	**2nd hospital day**	**6th hospital day**	**8th hospital day** *	**13th hospital day** †	**2 wk after discharge**	**3 wk after discharge**	**1 mo** **after discharge**	**2 mo after discharge**	**3 mo after discharge**
Aspartate aminotransferase (U/L)	10–40	360	412		477	196	246	91	32	28	27
Alanine aminotransferase (U/L)	10–40	566	417	457	497	324	399	231	63	43	26
Alkaline phosphatase (U/L)	40–125	94	81		86			123	102	97	75
γ-Glutamyl transferase (U/L)	15–85	38	28		38			159	115	61	40
Bilirubin (mg/dL)	<1	0.9	0.3	0.3	0.3	0.3	0.9	1.2	0.9		0.7
C-reactive protein (mg/L)	<3	100.9	155.4	69	63.6	16.2	6	1.5			
Serum amylase (U/L)	20–115	149	83	48	89						
Urine amylase (U/L)	<400	2566	736	100							

In summary, the patient was admitted with a clinical picture of hepatitis, pyuria, and colitis. She was put to a nil per os status except for her pills and was empirically started on intravenous ceftriaxone 2 g once a day, gentamicin 320 mg qd, ondansetron 8 mg twice a day (which led to immediate resolution of vomiting—she was able to drink water), and intravenous crystalloid fluids. After the first hospital day, abdominal pain and tenderness subsided, amylase values declined and oral feeding was subsequently started without complications.

Repeated urinalysis on third hospital day was normal, whereas stool and urine cultures from admission were negative. Flexible sigmoidoscopy was ordered without revealing any abnormalities. Fecal consistency restored from the fifth hospital day and thereafter; repeated microscopic stool examination on seventh hospital day was normal, without leucocytes. According to the above, working diagnoses of grade 1 ICPI-associated colitis and culture-negative urinary tract infection were made; antibiotic regimen was continued for 10 days in total. The possibility of ICPI renal toxicity as the cause of pyuria was not considered because urinalysis rapidly normalized without immunosuppressants and serum creatinine levels remained normal.

Even though the patient’s initial symptoms were improving, she developed febrile episodes high as 39.1^o^C from the third hospital day alongside continuous elevation of serum aminotransferases. Testing for antinuclear antibodies, extractable nuclear antigen, antisoluble liver antigen antibodies, smooth muscle antibodies, liver kidney microsome type 1 antibody, anti-liver cytosolic antigen type 1 antibodies, anti-F-actin antibody, viral hepatitis antigen and antibodies, ceruloplasmin, and serum protein electrophoresis did not reveal any abnormalities.

After meticulous exclusion of hospital-acquired infection, targeted release budesonide orally (12 mg/d) was administered with a working diagnosis of grade 3 ICPI-associated hepatotoxicity. Following budesonide initiation, fever subsided and AST and ALT values gradually declined. The patient was discharged and management continued on an outpatient basis. Aminotransferases returned to normal-near normal approximately 1 month after the first dose of budesonide (Table 1). After this point, daily dose was reduced by 3 mg every 2 weeks, with no clinical or biochemical relapse. There were no adverse events, except for mild bilateral leg edema that subsided when budesonide dose fell to 6 mg/d.

## 3. Discussion

Elevated ALT values have been observed following anti-CTLA-4 administration in 4% to 9% of patients and grade 3 or 4 hepatitis in 2% to 7% of the cases.^[12,13]^ Incidence of liver injury among patients treated with anti-PD-1 is 4% to 7% for any grade and 1% for grade ≥ 3, whereas hepatic toxicity during combined anti-CTLA-4 and anti-PD-1 treatment is much more frequent (19%–37% for any grade and 9%–16% for grade ≥ 3).^[13–15]^

We report the first patient managed with the ileal release form of budesonide as monotherapy for ICPI-related hepatitis. Use of budesonide in a similar case has been previously reported but in combination with systemic prednisolone (initial dose 1 mg/kg).^[16]^ American Gastroenterological Association Clinical Practice Update, as well as European Society for Medical Oncology and American Society of Clinical Oncology guidelines indicate an initial daily dose of intravenous prednisolone of 1 to 2 mg/kg for grade 3 hepatotoxicity.^[1–3]^ However, management recommendations for ICPI-induced liver injury rely more on clinical experience from AIH than on randomized trials in this setting.

Data from liver biopsies, interestingly, show that anti-PD-1 hepatitis, anti-CTLA-4 hepatotoxicity, and AIH present distinct histological findings. Liver injury from anti-PD-1 seems to be nonspecific, consisting of a lymphocytic (most often panlobular) pattern of hepatocellular injury along with portal inflammation.^[17,18]^ Features of anti-CTLA-4 hepatitis are different and include lobular hepatitis with poorly defined granulomas and fibrin ring granulomas, sinusoidal histiocytic infiltrates, and central vein endothelitis.^[17,19–21]^ On the other hand, histologic hallmarks of AIH like extensive plasma cell infiltration, hepatocellular rosettes and emperipolesis appear to be absent in ICPI-related hepatitis.

Concerning AIH, the initial recommended corticosteroid daily dosage from the 2015 European Association for the Study of the Liver update for AIH and 2019 American Association for the Study of Liver Diseases and Greek guidelines is 20 to 40 mg or 0.5 to 1 mg/kg prednisolone or its equivalent (classically 1 mg/kg of prednisolone if it is used as monotherapy).^[4–6]^ Although there are no studies to determine the equally efficient doses of budesonide and prednisolone for AIH, data from Crohn disease indicate that 9 mg of budesonide daily has similar efficacy to 40 mg of prednisolone.^[22,23]^ According to these findings, the equally efficient dose of budesonide to 1 mg/kg prednisolone could be approximately 15 mg/d for our patient. A regimen of 12 mg/d was nonetheless chosen because it is the highest dose given previously in the context of immunotherapy toxicity management; even higher doses have been safely used for the management of IgA nephropathy.^[24,25]^

On the other hand, it is known that aminotransferase levels, which is the main criterion determining ICPI hepatitis management according to the guidelines, do not reflect the grade of liver injury. For that reason, the publishment by De Martin et al^[17]^ was of great novelty in our opinion, relying treatment initiation on histology rather than on liver enzymes. That approach led to successful remission of grade ≥3 ICPI hepatitis in 6 of 16 patients having favorable liver histology only with cessation of immunotherapy. Great caution, however, is warranted when biopsy-driven watchful waiting is used for grade ≥3 ICPI hepatitis without signs of acute liver failure because it is not known yet when the peak of liver injury from ICPI appears. Μild histological findings in early stages may provide false reassurance, thus delaying corticosteroid initiation, which could possibly prevent an upcoming deterioration.

Overall, budesonide appears to be a potentially promising alternative for the management of ICPI hepatitis because of its mild adverse event profile, according to several studies for various indications. Of note, the statement of recent European guidelines on microscopic colitis was that oral budesonide does not significantly increase serious adverse events over placebo.^[26]^ This favorable profile makes budesonide a potential candidate for initial management of ICPI-induced liver injury without liver failure; biopsy could therefore be reserved for patients with grade ≥3 hepatotoxicity who do not respond or deteriorate within the first week of budesonide administration.

Another potential advantage of budesonide is that, most likely there is no need for dose tapering in order to reinitiate immunotherapy. When other glucocorticoids are used, dosage must be lowered to 10 mg/d of prednisolone or its equivalent to be able to resume treatment because systemically absorbed baseline steroids in higher doses compromise the effect of ICPI, regardless of tumor type.^[1–3,27]^ Although a recent single-center study suggests that underlying cancer related-symptoms managed with corticosteroids affect survival and not corticosteroids themselves, there is no doubt that these results need to be solidified by larger-scale, prospective trials.^[28]^ ICPI toxicity may also be enhanced; in the series studied by Pollack et al,^[29]^ patients receiving systemically acting corticosteroids during ICPI resumption presented higher rates of toxicity.

When budesonide is administered orally, even in a patient with overt colitis, which is not the case in the vast majority of patients having ICPI-related hepatotoxicity, systemic absorption will likely be no >21% measured in Crohn disease patients. Consequently, when a budesonide dosage of 12 mg is used as monotherapy and accepting that 9 mg of budesonide is equivalent to 40 mg of prednisolone, the tissues of systemic circulation (including tumor cells) are exposed to dosage smaller than (or equal to, in the worst-case scenario) 11 mg prednisolone equivalent. That element gives the ability for faster immunotherapy rechallenge if there is an indication for. However, it should be highlighted that the latter theoretical advantage of budesonide may be valid in selected patients without residual tumor burden in tissues affected by enterohepatic circulation (like liver and intestine) and without the aforementioned factors influencing drug’s first-pass hepatic metabolism. Further studies with more patients should assess better the efficacy of budesonide in ICPI liver injury management, thus validating or refuting these findings.

## 4. Conclusion

Available evidence indicates that corticosteroid treatment is a necessary intervention in the majority of cases with grade ≥3 ICPI hepatitis. However, the patients who will benefit from corticotherapy, as well as the route of administration and daily dose have not yet been clarified by large-scale, randomized trials. This case report highlights that budesonide may be a potential option for ICPI hepatotoxicity without liver failure; in order to minimize the risk of corticosteroid-related adverse events, rechallenge with ICPI faster and possibly eliminate the need for liver biopsy in responders. Clinical trials assessing the role of budesonide in this setting should be conducted in the future.

## Author contributions

Eleftheriotis G: conceptualization, methodology, validation, writing – original draft; Skopelitis E: validation, supervision, writing – review & editing.

## Footnotes

* Day of budesonide initiation.

† On discharge.

## References

[1] Haanen JBAG Carbonnel F Robert C. Management of toxicities from immunotherapy: ESMO Clinical Practice Guidelines for diagnosis, treatment and follow-up. Ann Oncol. 2017;28(Suppl_4):iv119–42. 28881921 10.1093/annonc/mdx225

[2] Brahmer JR Lacchetti C Schneider BJ. Management of immune-related adverse events in patients treated with immune checkpoint inhibitor therapy: American Society of Clinical Oncology Clinical Practice Guideline. J Clin Oncol. 2018;36:1714–68. 29442540 10.1200/JCO.2017.77.6385 PMC6481621

[3] Dougan M Wang Y Rubio-Tapia A. AGA clinical practice update on diagnosis and management of immune checkpoint inhibitor (ICI) colitis and hepatitis: expert review. Gastroenterology. 2021;160:1384–93. 33080231 10.1053/j.gastro.2020.08.063

[4] Manns MP Lohse AW Vergani D. Autoimmune hepatitis—update 2015. J Hepatol. 2015;62:S100–11. 25920079 10.1016/j.jhep.2015.03.005

[5] Dalekos GN Koskinas J Papatheodoridis GV. Hellenic association for the study of the liver clinical practice guidelines: autoimmune hepatitis. Ann Gastroenterol. 2019;32:1–23. 30598587 10.20524/aog.2018.0330 PMC6302199

[6] Mack CL Adams D Assis DN. Diagnosis and management of autoimmune hepatitis in adults and children: 2019 practice guidance and guidelines from the American Association for the Study of Liver Diseases. Hepatology. 2020;72:671–722. 31863477 10.1002/hep.31065

[7] Efe C Hagström H Ytting H. Efficacy and safety of mycophenolate mofetil and tacrolimus as second-line therapy for patients with autoimmune hepatitis. Clin Gastroenterol Hepatol. 2017;15:1950–1956.e1. 28603052 10.1016/j.cgh.2017.06.001

[8] Edsbäcker S Andersson T. Pharmacokinetics of budesonide (Entocort™ EC) capsules for Crohn’s disease. Clin Pharmacokinet. 2004;43:803–21. 15355126 10.2165/00003088-200443120-00003

[9] Manns MP Woynarowsky M Kreisel W. Budesonide induces remission more effectively than prednisolone in a controlled trial of patients with autoimmune hepatitis. Gastroenterology. 2010;139:1198–206. 20600032 10.1053/j.gastro.2010.06.046

[10] Edsbäcker S Bengtsson B Larsson P. A pharmacoscintigraphic evaluation of oral budesonide given as controlled-release (Entocort) capsules. Aliment Pharmacol Ther. 2003;17:525–36. 12622761 10.1046/j.1365-2036.2003.01426.x

[11] Edsbäcker S Larsson P Höglund P. Systemic exposure and availability of orally administered budesonide capsules (Entocort™ EC) vs methylprednisolone [abstract]. Am J Gastroenterol. 2002;97:S262.

[12] O’Day SJ Maio M Chiarion-Sileni V. Efficacy and safety of ipilimumab monotherapy in patients with pretreated advanced melanoma: a multicenter single-arm phase II study. Ann Oncol. 2010;21:1712–7. 20147741 10.1093/annonc/mdq013

[13] Wolchok JD Chiarion-Sileni V Gonzalez R. Overall survival with combined nivolumab and ipilimumab in advanced melanoma. N Engl J Med. 2017;377:1345–56. 28889792 10.1056/NEJMoa1709684 PMC5706778

[14] Mok TSK Wu YL Kudaba I. Pembrolizumab versus chemotherapy for previously untreated, PD-L1-expressing, locally advanced or metastatic non-small-cell lung cancer (KEYNOTE-042): a randomised, open-label, controlled, phase 3 trial. Lancet. 2019;393:1819–30. 30955977 10.1016/S0140-6736(18)32409-7

[15] Tawbi HA Forsyth PA Algazi A. Combined nivolumab and ipilimumab in melanoma metastatic to the brain. N Engl J Med. 2018;379:722–30. 30134131 10.1056/NEJMoa1805453 PMC8011001

[16] Ziemer M Koukoulioti E Beyer S. Managing immune checkpoint-inhibitor-induced severe autoimmune-like hepatitis by liver-directed topical steroids. J Hepatol. 2017;66:657–9. 27908801 10.1016/j.jhep.2016.11.015

[17] De Martin E Michot JM Papouin B. Characterization of liver injury induced by cancer immunotherapy using immune checkpoint inhibitors. J Hepatol. 2018;68:1181–90. 29427729 10.1016/j.jhep.2018.01.033

[18] Zen Y Yeh MM. Hepatotoxicity of immune checkpoint inhibitors: a histology study of seven cases in comparison with autoimmune hepatitis and idiosyncratic drug-induced liver injury. Mod Pathol. 2018;31:965–73. 29403081 10.1038/s41379-018-0013-y

[19] Kleiner DE Berman D. Pathologic changes in ipilimumab-related hepatitis in patients with metastatic melanoma. Dig Dis Sci. 2012;57:2233–40. 22434096 10.1007/s10620-012-2140-5 PMC3792485

[20] Johncilla M Misdraji J Pratt DS. Ipilimumab-associated hepatitis: clinicopathologic characterization in a series of 11 cases. Am J Surg Pathol. 2015;39:1075–84. 26034866 10.1097/PAS.0000000000000453

[21] Everett J Srivastava A Misdraji J. Fibrin ring granulomas in checkpoint inhibitor-induced hepatitis. Am J Surg Pathol. 2017;41:134–7. 27792061 10.1097/PAS.0000000000000759

[22] Rutgeerts P Löfberg R Malchow H. A comparison of budesonide with prednisolone for active Crohn’s disease. N Engl J Med. 1994;331:842–5. 8078530 10.1056/NEJM199409293311304

[23] Campieri M Ferguson A Doe W. Oral budesonide is as effective as oral prednisolone in active Crohn’s disease. The Global Budesonide Study Group. Gut. 1997;41:209–14. 9301500 10.1136/gut.41.2.209 PMC1891473

[24] Hughes MS Molina GE Chen ST. Budesonide treatment for microscopic colitis from immune checkpoint inhibitors. J ImmunoTher Cancer. 2019;7:292. 31699151 10.1186/s40425-019-0756-0 PMC6839080

[25] Fellström BC Barratt J Cook H. Targeted-release budesonide versus placebo in patients with IgA nephropathy (NEFIGAN): a double-blind, randomised, placebo-controlled phase 2b trial. Lancet. 2017;389:2117–27. 28363480 10.1016/S0140-6736(17)30550-0

[26] Miehlke S Guagnozzi D Zabana Y. European guidelines on microscopic colitis: United European Gastroenterology (UEG) and European Microscopic Colitis Group (EMCG) statements and recommendations. United European Gastroenterol J. 2021;9:13–37. 10.1177/2050640620951905 PMC8259259 33619914

[27] Drakaki A Dhillon PK Wakelee H. Association of baseline systemic corticosteroid use with overall survival and time to next treatment in patients receiving immune checkpoint inhibitor therapy in real-world US oncology practice for advanced non-small cell lung cancer, melanoma, or urothelial carcinoma. Oncoimmunology. 2020;9:1824645. 33101774 10.1080/2162402X.2020.1824645 PMC7553559

[28] Gaucher L Adda L Séjourné A. Impact of the corticosteroid indication and administration route on overall survival and the tumor response after immune checkpoint inhibitor initiation. Ther Adv Med Oncol. 2021;13:1758835921996656. 33717227 10.1177/1758835921996656 PMC7923985

[29] Pollack MH Betof A Dearden H. Safety of resuming anti-PD-1 in patients with immune-related adverse events (irAEs) during combined anti-CTLA-4 and anti-PD1 in metastatic melanoma. Ann Oncol. 2018;29:250–5. 29045547 10.1093/annonc/mdx642 PMC5834131

